# Impact of waitlist weight change on outcomes in heart transplant recipients: a UNOS database analysis

**DOI:** 10.1007/s11748-024-02078-y

**Published:** 2024-10-03

**Authors:** Melissa A. Austin, Danial Ahmad, Jake L. Rosen, Matthew P. Weber, Indranee Rajapreyar, Jesus Eduardo Rame, Rene J. Alvarez, John W. Entwistle, Howard T. Massey, Vakhtang Tchantchaleishvili

**Affiliations:** 1 Division of Cardiac Surgery, Thomas Jefferson University, 1025 Walnut Street, College Building, Suite 607, Philadelphia, PA 19107 USA; 2 Division of Cardiology, Thomas Jefferson University, Philadelphia, PA USA

**Keywords:** Heart failure, Heart transplantation, Database, Weight loss, Weight gain, Cardiac cachexia, Obesity

## Abstract

**Background:**

While the effect of pre-transplant weight on patient outcomes following heart transplantation (HTx) has previously been studied, data regarding the impact of dynamic weight change prior to HTx are extremely limited.

**Objectives:**

We sought to elucidate the interaction between HTx listing weight and weight change while waitlisted, and explore how that interaction impacts post-HTx survival in a continuous manner.

**Methods:**

Adult patients listed for HTx from 1987 to 2020 were identified from UNOS database. Three-dimensional restricted cubic spline analysis explored post-HTx survival relative to both changes in BMI/weight and BMI at time of HTx listing. Continuous predictor variables were analyzed with Cox proportional hazards method.

**Results:**

9,628 included patients underwent HTx. Median recipient age was 55 [IQR 46–62] years, and 21% were females. 53% of patients lost while 47% gained weight on the waitlist. Median BMI (27.6 kg/m^2^ [24.3–31.3] vs. 27.4 kg/m^2^ [24.2–30.9], paired *p* < 0.001) and weight (84.8 kg [73.0–98.0] kg vs. 84.4 kg [72.6–96.6], *p* < 0.001) were similar at listing and transplant. One-year survival was 89.3%. Weight loss over 3 BMI points or 10 kg was associated with higher hazard of death irrespective of listing BMI. In non-obese patients, some weight gain (1–4 BMI points or 5–15 kg) was associated with improved survival. In cachectic patients (BMI < 18.5), failure to gain weight was associated with worse survival.

**Conclusions:**

Impact of weight change varies depending on listing BMI. While a survival benefit is seen in non-obese patients who gain some weight, significant weight loss is associated with poorer survival.

**Supplementary Information:**

The online version contains supplementary material available at 10.1007/s11748-024-02078-y.

## Introduction

Over 3400 people are currently waiting for a heart transplant in the United States [1], with median waitlist waiting time ranging from 2 to 24 months [2]. Optimizing patients for heart transplant surgery during this waiting period is central to improving post-transplant outcomes, and patient weight might represent a key factor to optimize. Current International Society of Heart and Lung Transplantation (ISHLT) guidelines recommend weight loss prior to cardiac transplantation listing for patients with BMI > 35 kg/m [2, 3]. This is based on prior studies demonstrating that patients with class II/III obesity (BMI > 35) have significantly higher morbidity and mortality following heart transplant compared to obesity class I, overweight and normal weight patients [4]. More recent studies have suggested variable BMI cutoff recommendations, but generally conclude increased post-transplant mortality over the BMI range of 35–38 [5, 6]. Further, diminished post-heart transplant survival has also been reported in underweight patients (BMI < 18.5) [4, 7]. While encouraging weight loss prior to heart transplant listing through measures such as bariatric surgery has been discussed in previous studies [8, 9], data on the impact of losing weight while already listed are scarce. Moreover, published data are lacking on the effect of gaining weight while on the waitlist.

Previous studies have demonstrated that significant weight loss (> 5% of body weight) generally portends a worse prognosis for obese patients with heart failure [10, 11]; however, there are currently no large-scale studies describing the impact of weight change on post-heart transplant survival in waitlisted patients. While weight may be feasibly modifiable in many heart failure patients, it is first necessary to understand how weight change impacts post-heart transplant survival prior to recommending its modification in either direction.

Thus, we first sought to elucidate the association between post-heart transplant survival and both listing BMI and waitlist weight change in a continuous manner. Weight change was analyzed in terms of BMI and kilograms to examine both the relative and absolute relationships. Our decision to explore relationships in a continuous manner was influenced by previous studies which identified problems innate to the dichotomization of continuous variables, including the loss of statistical power to identify a relationship as well as underestimation of variation within categorized groups [12]. In order to investigate if the survival impact of weight change can be generalized to all initial BMIs, we also aimed to analyze how the interaction between listing BMI and waitlist weight change impacts post-transplant survival.

## Materials and methods

### Study population and database utilization

This study was exempt from institutional review board review. Adult patients (> 18 years of age) who received a primary, heart-only transplant between October 1987 and June 2020 were selected from the UNOS database. Data from UNOS thoracic dataset were combined using transplant case-identifying numbers in order to create a single dataset. Patients chosen for analysis were limited to those with documented weight at both listing and time of transplant, and survival data at 1 year post-transplantation.

### Missing data

While patient weight at time of listing is well documented in the UNOS database, weight at transplant is more likely to be either missing or represent a value carried over from the listing weight data without remeasuring the patient. We found a large number of patients with identical weights at listing and at transplant (to the third decimal place), which were presumed to be carried over and thus were considered missing data. After applying this selection criteria, 52,818 of the original 63,065 initial adult heart transplant patients in the UNOS database had missing weight data (Supplemental Fig. 1). Considering the large proportion (> 80%) of missing data, we did not impute missing data and only analyzed complete cases. The extent of missing data also motivated our inclusion of the broadest possible time frame (past 35 years) of available data.

### Handling of outliers

The boxplot outlier detection model (identifies data points which fall outside of [Quartile 1 − 1.5 IQR]—[Quartile 3 + 1.5 IQR]) was used to exclude patients with waitlist BMI or weight change values outside of the calculated boxplot outlier “fence.” This methodology removed about 600 more patients from the analysis (Supplemental Fig. 1).

### Descriptive statistical analysis

Patient baseline characteristics and clinical data were obtained and analyzed using summary statistics. Categorical variables were reported as percentages and continuous data were reported as medians and interquartile ranges (IQR). Body mass index (BMI) and weight (in kilograms) at waitlist listing and at transplantation were compared using paired Wilcoxon signed-rank tests, with *p* value < 0.05 considered statistically significant. Box plots were used to depict both patient-level and summary statistics for BMI change. Effect size was calculated as Cohen’s d to analyze the size of the overall difference between BMI at listing and at transplant for the patient cohort (*d* > 0.2 considered significant).

### Tensor spline analysis

Relationships between listing BMI or weight variables and patient survival at 1 year were explored in a continuous manner, without arbitrary categorization, using restricted cubic spline (RCS) analysis [13]. The number of knots in each spline model was determined using the lowest value of Akaike information criterion (AIC), which maximizes the model’s likelihood ratio [14]. Cox-proportional hazard model was used for survival analysis, and is reported as log of the hazard ratio.

Next, the continuous spectrum of listing BMI and BMI/weight change variables were simultaneously plotted against one another using a three-dimensional tensor spline analysis and this was used to explore how their interaction was associated with patient survival at 1 year.

The three-dimensional RCS models were depicted as heat maps describing relative hazard of death at 1 year following transplant [blue (best) to red (worst)] at various intersections of weight change and listing BMI. R software, version 3.6.0 (R Foundation for Statistical Computing, Vienna, Austria) was used for all data analysis and visualization (packages: rms [15]).

## Results

### Baseline characteristics

There were 63,065 adult patients who underwent primary, single organ heart transplantation queried from the UNOS thoracic dataset. After screening with the selection criteria, 9628 patients were included for analysis. The median recipient age was 55 years [IQR 46–62] and 21% (1993/9628) were female. While the majority of the patients identified as White (70.4%), 18.9% (1819/9628) identified as Black, and 7.6% (733/9628) as Hispanic. 27.3% (2632/9628) of patients had undergone prior cardiac surgery. The median time on the heart transplant waitlist was 233 days [IQR 90–529]. The most common primary cardiac diagnoses were idiopathic dilated cardiomyopathy in 35.7% (3433/9628) of patients, ischemic cardiomyopathy in 33.5% (3223/9628), and coronary artery disease in 6.0% (578/9628). Additional baseline characteristics can be found in Table 1.

**Table 1:** Pre-transplant baseline characteristics and post-transplant patient outcomes

Baseline characteristics	
Variable	Total (*n* = 9628)
Recipient age (years), median [IQR]	55 [46, 62]
Male recipient, *n* (%)	7635 (79.3)
Recipient height (cm), median [IQR]	175.3 [168.0, 182.9]
Recipient weight (kg), median [IQR]	84.4 [72.6, 97.5]
Smoker, *n* (%)	3492 (36.3)
Prior cardiac surgery	2632 (27.3)
Ethnicity, *n* (%)	
White	6780 (70.4)
Black	1819 (18.9)
Hispanic	733 (7.6)
Asian	175 (1.8)
Cerebrovascular disease, *n* (%)	467 (4.9)
Diabetes, *n* (%)	
Type I	122 (1.3)
Type II	1837 (19.1)
Donor-recipient match level, *n* (%)	
Identical	8509 (88.4)
Compatible	1118 (11.6)
Incompatible	1 (0.0)
Dialysis prior to transplant, *n* (%)	261 (2.7)
On life support, *n* (%)	5126 (53.2)
Total days on waitlist, median [IQR]	233 [90, 529]
Primary cardiac diagnosis, *n* (%)	
Idiopathic dilated cardiomyopathy	3433 (35.7)
Ischemic dilated cardiomyopathy	3223 (33.5)
Coronary artery disease	578 (6.0)
Postoperative outcomes	
Length of stay (days), median [IQR]	15 [10, 23]
Length of follow-up, year [IQR]	4.04 [1.36, 8.86]
One-year survival, *n* (%)	8594 (89.3)
Stroke, *n* (%)	291 (3.0)
Dialysis after transplant, *n* (%)	1021 (10.6)
Pacemaker, *n* (%)	312 (3.2)
Most common causes of death, *n* (%)	
Cardiac arrest	302 (3.1)
Pancreatitis	286 (3.0)
Bacterial septicemia	182 (1.9)

### Postoperative characteristics and outcomes

Median length of stay in the hospital following heart transplantation was 15 days [IQR 10–23]. One-year survival was 89.3% (8594/9628). Post-transplant complications included stroke in 3.0% (291/9628) and pacemaker placement in 3.2% (312/9628) of patients. Additional postoperative outcomes can be found in Table 1.

### Weight and BMI change

**Fig. 1:**
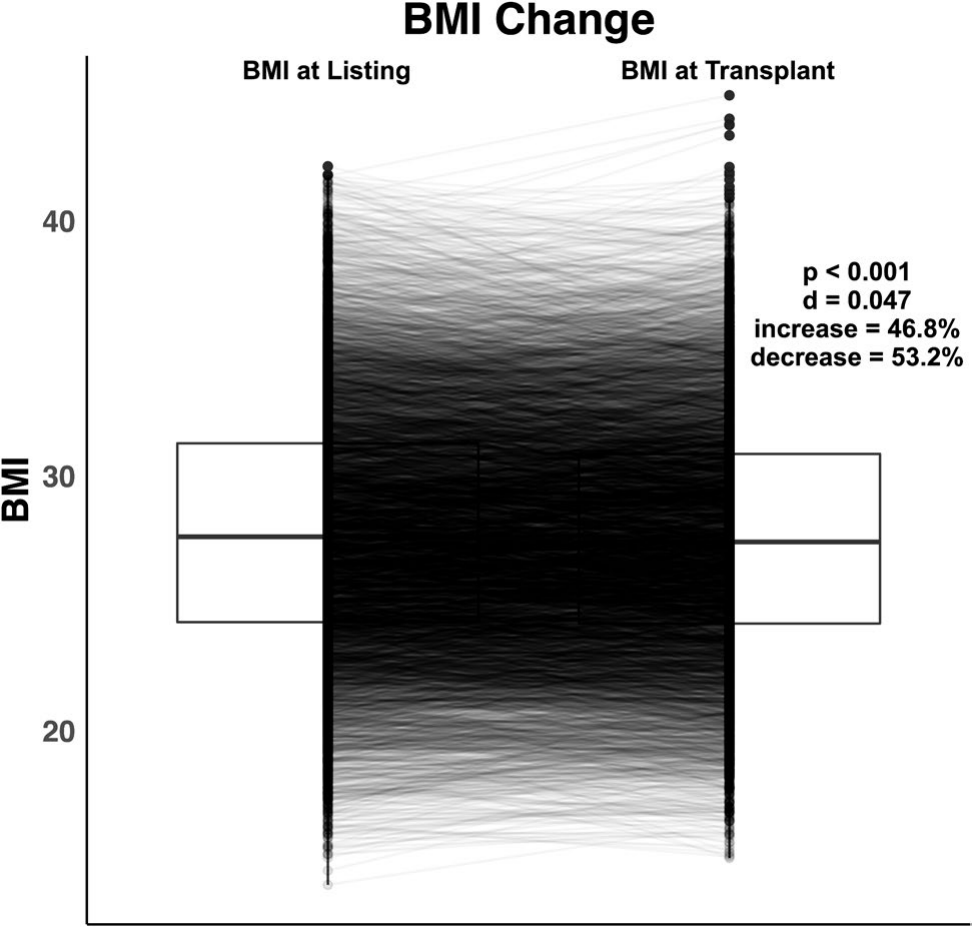
BMI Change Between Listing and Transplant. Box plots depicting patient BMI at listing and at time of heart transplant. Each line between the box plots represents individual patient BMI change. Paired analysis demonstrated significantly different patient BMI at listing and transplant (*p* < 0.001). However, there was a negligible difference in overall median BMI of the patient cohort as demonstrated by the effect size (Cohen’s *d* < 0.2). (*BMI* body max index)

Figure 1 depicts patient-level BMI change between listing and transplant, with 53% of patients losing weight and 47% gaining weight while on the waitlist. The median patient weight at listing was 84.8 kg [IQR 73.0–98.0] and at transplant was 84.4 kg [IQR 72.6–96.6] (*p* < 0.001 with paired analysis). The median patient BMI at listing was 27.6 [IQR 24.3–31.3] and at transplant was 27.4 [IQR 24.2–30.9] (*p* < 0.001). While the patient-level, paired analysis demonstrated significant weight change, the effect size (Cohen’s *d* < 0.05) was small for median overall weight change (0.0 kg [IQR 5.5–4.1]) and BMI change (− 0.1 kg/m^2^ [IQR 1.8–1.4]).

### Weight change and survival

**Fig. 2:**
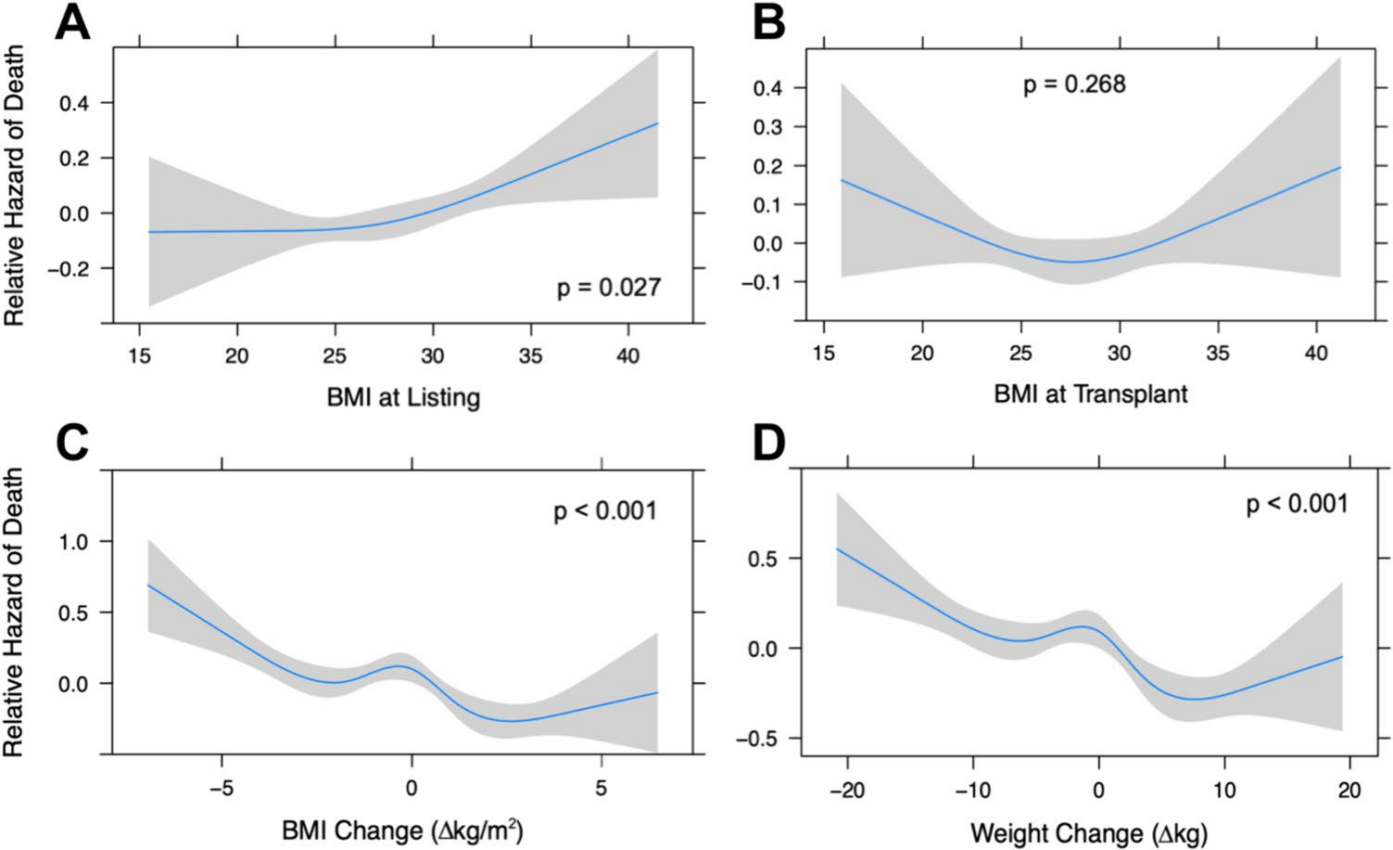
Impact of Weight and Weight Change on Patient Survival. Restricted cubic spline analysis describing the association between patient survival at 1 year (relative hazard of death) following heart transplant and the following continuous variables: BMI at listing (**A**), BMI at transplant (**B**), BMI change (**C**), and Weight change (**D**). (*BMI* body max index)

The restricted cubic spline models in Fig. 2 depict the relative hazard of death at 1 year for the continuous spectrum of BMI at listing (Panel A), BMI at transplant (Panel B), BMI change between listing and transplant (Panel C) and weight change between listing and transplant (Panel D). Our analysis demonstrates that survival was significantly associated with BMI at listing (*p* = 0.027), BMI change (*p* < 0.001) and weight change (*p* < 0.001); however, BMI at the time of transplant was not significantly associated with survival (*p* = 0.268).

**Fig. 3:**
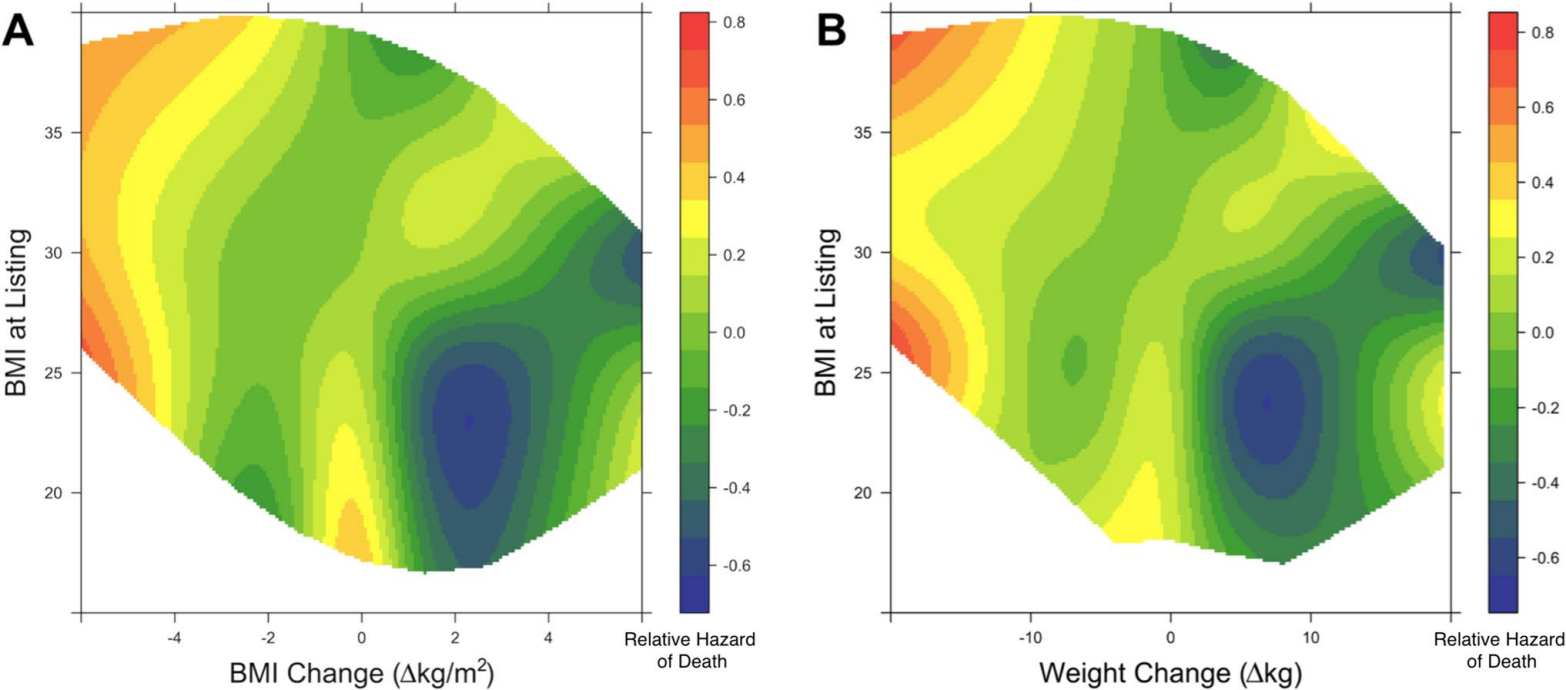
Impact of waitlist weight change in heart transplant recipients varies with listing BMI. Three-dimensional restricted cubic spline analysis depicting post-heart transplant survival relative to both changes in BMI/weight and BMI at time of heart transplant listing. Heatmap colors represent relative hazard of death at 1 year [blue (best) to red (worst)]. (*BMI* body max index)

To simultaneously explore overall post-heart transplant survival at 1 year relative to both changes in BMI/weight and weight at time of heart transplant listing, three-dimensional RCS analysis was performed. As demonstrated by Fig. 3, weight loss greater than 3 BMI points (Panel A) or 10 kg (Panel B) was associated with higher relative hazard of death. This finding appears to be irrespective of listing BMI. In non-obese patients (BMI < 30), some weight gain (1–4 BMI points or 5–15 kg) was associated with improved survival. Further, in cachectic patients (BMI < 20), failure to gain weight was associated with worse survival.

## Discussion

Despite previous evidence demonstrating the association between static pre-transplant BMI > 35 or BMI < 18.5 and worse patient outcomes following heart transplantation [4–7], data on the impact of dynamic weight change (weight loss or gain) prior to heart transplantation are extremely limited. While there are some data regarding weight change prior to other solid-organ transplantation, the evidence is limited to weight loss and conflicting in conclusions. For example, Harhay et al. demonstrated significantly higher graft loss and mortality in kidney transplantation patients who lost > 10% of weight between listing and transplant [16]. However, a follow-up study concluded that only unintentional weight loss was associated with worse outcomes [17]. In contrast, studies investigating the impact of weight loss prior to lung transplantation reported improved survival directly proportional to the percentage of weight lost between listing and transplant [18, 19]. The conflicting nature of data describing the survival impact of weight loss for lung and kidney transplant patients limits our ability to translate these study conclusions to heart transplant patients, and thus highlights the need for heart transplant-specific data. Recently presented data from Hess et al. analyzed post-heart transplant outcomes in patients stratified into stable weight, weight loss and weight gain (≥ 5%) [20]. They found that the weight loss cohort was associated with lowest 90-day and 1-year post-transplant survival. To our knowledge, the current study is the first to look at the association between heart transplant patient survival and weight change while on the transplant waitlist in a continuous, non-stratified manner.

In our initial analysis of the impact of BMI on patient survival, we utilized RCS models to analyze relative hazard of death over the continuous spectrum of patient BMI values at listing and transplant. This analysis demonstrated that BMI at listing (Fig. 2A) was significantly associated with post-transplant survival (*p* = 0.027), with BMI > 35 associated with increased relative hazard of death post-transplant. While BMI at transplant was not significantly associated with post-transplant survival (*p* = 0.268), the concave shape of the spline more closely resembles previous studies demonstrating significant association between both BMI extremes at time of transplant (underweight or class II/III obesity) and worse post-transplant survival [4–7]. The degree of missing data in the current study likely impacted the significance level of the association between BMI at transplant and post-transplant survival. Additionally, the continuous analysis approach of the current study allowed us to avoid arbitrary stratification, and likely played a role in the discrepancies between our findings and previous study conclusions.

In our initial analysis of the impact of BMI/weight change on patient survival, we utilized RCS curves to analyze relative hazard of death over the continuous spectrum of BMI changes (Fig. 2C) and weight changes (Fig. 2D) in our patient cohort. The figures describe a potential protective effect of weight gain (up to about 3 BMI points or 10 kg). This finding aligns well with the previously reported “obesity paradox,” which describes improved survival seen in obese patients with moderate to severe heart failure despite obesity’s contributory role in cardiovascular disease development [21–23]. One popularized theory behind the obesity paradox is that elevated BMI might provide an increased metabolic reserve in the setting of a catabolic disease (HF) [23]. This theory might similarly explain the protective effect of weight gain prior to transplant surgery, as the body’s stress response to surgery is catabolism of stored body fuels [24]. On the opposite side of the continuous spectrum, the RCS analyses in Fig. 2C and D also demonstrate increased relative hazard of death with weight loss greater than about 5 BMI points or 10 kg. It is plausible that the diminishing stores of body ‘fuel’ previously mentioned might contribute to the association between significant weight loss and worse post-transplant survival. However, it is more likely that this is a multifactorial relationship also influenced by factors such as aggressive diuresis in overloaded heart failure patients and cachexia due to body wasting and poor nutrition seen in critical illness.

We next aimed to investigate if the current study’s aforementioned associations between weight change and survival could be generalized to patients of any initial BMI at time of listing. We were able to investigate this, without arbitrary categorization, via three-dimensional tensor spline analysis (Fig. 3). One major conclusion from Fig. 3 is that in non-obese patients (BMI < 30), some weight gain (up to ~ 15 kg or 4 BMI points) was potentially protective. While weight gain in obese patients (BMI > 30) was not necessarily protective, it was also not associated with increased hazard of death. Additional studies are required to further investigate the physiology underlying this relationship.

Another major conclusion from Fig. 3 is that weight loss more than about 10 kg or 3 BMI points was associated with worse survival, regardless of initial BMI at listing. Weight loss greater than 10 kg or 3 BMI points is generally significant; thus, the exercise and diet restrictions associated with severe cardiac dysfunction raise the question of weight loss intentionality. Whenever significant weight loss occurs in patients with heart failure, the possibility of cardiac cachexia should be explored. While the exact definition of cardiac cachexia is highly debated, there is a general consensus that it is a complex syndrome describing weight loss due to body-wasting, and is associated with poor prognosis in heart failure patients [25]. Similar to cachexia seen in other terminal stages of disease, such as in cancer, cachexia in heart failure usually signifies severe bodily dysfunction that cannot be explained by simple nutritional deficiency [25]. Thus, cardiac cachexia may very well play a role in the association between significant weight loss and worse survival, regardless of initial BMI, as reported in the current study. Another possible explanation is that patients in severe heart failure who are fluid overloaded might lose significant weight through aggressive diuresis.

The last major conclusion from Fig. 3 is that cachectic patients (BMI < 18.5) who fail to gain weight seem to have worse post-transplant survival at 1 year. Further, it appears that some weight gain might be associated with improved survival in these underweight patients. While there are no currently approved treatments for the indication of cardiac cachexia, nutritional support and prevention of further weight loss is deemed central to cachexia management [25]. Thus, the improved survival with some weight gain compared to the diminished survival with weight loss reported in this study may be related to nutritional deficiencies which were likely improved with weight gain and worsened with weight loss. Considering weight gain can have various etiologies, commonly fluid fluctuations or increase in adipose tissue for heart failure patients, other factors may also play currently unknown roles in this relationship.

## Limitations

This study was limited due to several factors. First, an observational, retrospective study is always subject to selection bias. Additionally, database analyses are limited by missing data and lack of granular data, which precludes the differentiation of intentional versus unintentional weight changes in our cohort of patients. Furthermore, it limits our understanding of the quality of this weight change, prohibiting the differentiation between fluid shifts versus tissue loss/gain. Our analysis also did not account for the time over which weight change occurred, and this temporal relationship may play a confounding role. Lastly, while survival is always subject to possible confounding factors, this study used strictly weight and BMI as predictor variables for survival.

## Conclusions

The impact of weight change while on the heart transplant waitlist varies depending on listing BMI. While survival benefit is seen in non-obese patients who gain some weight, significant weight loss is generally associated with poorer survival regardless of initial BMI. Future prospective analysis regarding the precise impact of pre-transplant weight change on heart transplant outcomes, and the associated physiology, would be beneficial to assist in heart transplant recipient patient counseling.

## Supplementary Information

Below is the link to the electronic supplementary material.

Supplementary file1 (DOCX 150 kb)
