# Supplementary material for: Impact of waitlist weight change on outcomes in heart transplant recipients: a UNOS database analysis
Source: Gen Thorac Cardiovasc Surg. 2024 Oct 3;73(5):336–42. doi: 10.1007/s11748-024-02078-y (PMC11993457; doi:10.1007/s11748-024-02078-y)

**Title:** Impact of Waitlist Weight Change on Outcomes in Heart Transplant Recipients: A UNOS Database Analysis

**Authors:** Melissa A. Austin MD^a^, Danial Ahmad MD^a^, Jake L. Rosen BS^a^, Matthew P. Weber MD^a^, Indranee Rajapreyar MD^b^, Jesus Eduardo Rame MD^b^, Rene J. Alvarez MD^b^, John W. Entwistle MD, PhD^a^, Howard T. Massey MD^a^, Vakhtang Tchantchaleishvili MD^a^

**Institutions:**

^a^Division of Cardiac Surgery, Thomas Jefferson University, Philadelphia, Pennsylvania, USA

^b^Division of Cardiology, Thomas Jefferson University, Philadelphia PA

**Supplemental Figure 1:** Flow chart depicting the data selection process


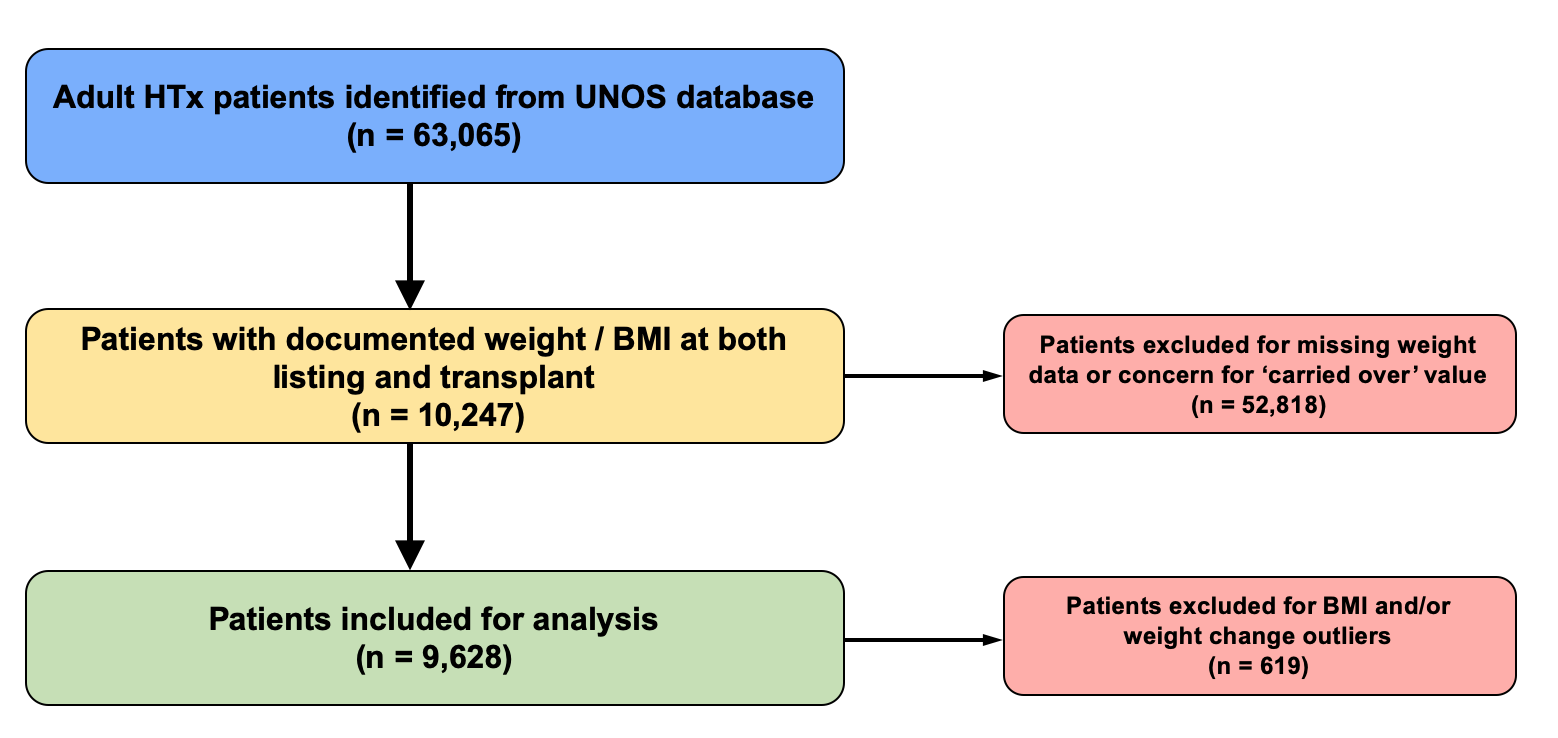

Supplement: Supplementary file 1 — Supplementary file1 (DOCX 150 kb) [file 11748_2024_2078_MOESM1_ESM.docx]
